# Loss of Lymphatic IKKα Disrupts Lung Immune Homeostasis, Drives BALT Formation, and Protects against Influenza

**DOI:** 10.4049/immunohorizons.2400047

**Published:** 2024-07-15

**Authors:** Michelle D. Cully, Julianne E. Nolte, Athena Patel, Andrew E. Vaughan, Michael J. May

**Affiliations:** Department of Biomedical Sciences, University of Pennsylvania School of Veterinary Medicine, Philadelphia, PA

## Abstract

IκB kinase (IKK)α controls noncanonical NF-κB signaling required for lymphoid organ development. We showed previously that lymph node formation is ablated in *Ikkα^Lyve-1^* mice constitutively lacking IKKα in lymphatic endothelial cells (LECs). We now reveal that loss of IKKα in LECs leads to the formation of BALT in the lung. Tertiary lymphoid structures appear only in the lungs of *Ikkα^Lyve-1^* mice and are not present in any other tissues, and these highly organized BALT structures form after birth and in the absence of inflammation. Additionally, we show that *Ikkα^Lyve-1^* mice challenged with influenza A virus (IAV) exhibit markedly improved survival and reduced weight loss compared with littermate controls. Importantly, we determine that the improved morbidity and mortality of *Ikkα^Lyve-1^* mice is independent of viral load and rate of clearance because both mice control and clear IAV infection similarly. Instead, we show that IFN-γ levels are decreased, and infiltration of CD8 T cells and monocytes into *Ikkα^Lyve-1^* lungs is reduced. We conclude that ablating IKKα in LECs promotes BALT formation and reduces the susceptibility of *Ikkα^Lyve-1^* mice to IAV infection through a decrease in proinflammatory stimuli.

## Introduction

Tertiary lymphoid structures (TLSs) are unencapsulated lymphoid organs that arise de novo in tissues in a wide range of infectious, inflammatory, and autoimmune diseases, including some cancers (1, 2). TLSs share many of the cellular and structural characteristics of the developmentally defined secondary lymphoid organs, including lymph nodes (LNs), and support the development of local immune responses. In autoimmune and chronic inflammatory diseases, TLSs can exacerbate disease; however, in some infections, cancers, and organ transplants, TLSs support protective immune responses and disease resolution (3–8). Due to these context-dependent roles of TLSs, understanding the specific cells and mechanisms that control their formation and maintenance and determining how distinct TLSs function in separate diseases to drive or reduce pathology are rapidly growing areas of intense investigation (1, 3–5).

Induced BALT (iBALT) is a type of TLS that forms in the perivascular and peribronchiolar spaces of the lung in response to infection, inflammation, and pulmonary damage (4). Like other TLSs, the function of iBALT is context dependent, exacerbating smoking-induced chronic obstructive pulmonary disease (3, 4) but supporting the draining LNs to provide protective immune responses to respiratory infections (6–13). Remarkably, antiviral immune responses generated within iBALT alone are sufficient to provide immunity in the lung (6, 9, 14). Moreover, iBALT formed in response to prior respiratory virus infection supports secondary immune responses following subsequent exposure (15), and LPS-induced iBALT in neonates provides accelerated immune responses to respiratory viruses in adult mice (11, 12). Collectively, these studies indicate that iBALT can support local immune responses to respiratory viruses, and, consequently, driving beneficial formation of these structures while avoiding pathogenesis represents a potentially powerful prophylactic approach to promote immunity in the lung (10, 16).

Although the precise mechanisms of TLS formation remain to be fully elucidated, extensive research has revealed mechanistic overlap with the cells and signals that control normal LN development. An overall model proposed by da Graça et al. involves three major steps of TLS formation: local fibroblast activation, recruitment of immune cells, and maturation of the TLS (1). Driving this process is ligation of the lymphotoxin-β receptor (LTβR) and activation of noncanonical NF-κB in tissue fibroblasts that induces expression of CXCL13, CCL21, and CCL19, chemokines that recruit immune cells, including APCs and lymphocytes, to the TLS site (1). Notably, overexpression of CXCL13 alone is sufficient to induce TLSs (17), and mice deficient in all three chemokines cannot form TLSs (7). Furthermore, although LTβR ligation is sufficient to induce TLS development, LTβR-independent mechanisms have also been described (11), and *Ltβr^−/−^* mice form T and B cell aggregates in the lungs that resemble iBALT (18). Maturation of TLSs involves the formation of a B cell follicle with follicular dendritic cells (DCs) that can support germinal center (GC) reactions, the development of high endothelial venules (HEVs) that facilitate naive lymphocyte recruitment, and the formation of proximal lymphatic networks (1, 2). The role of lymphatic vessels in the overall development of TLSs remains very poorly defined; however, patients with lymphatic malformations develop TLSs in the lesion area, and loss of lung lymphatic integrity in mice leads to iBALT formation (19, 20). These collective findings suggest that disruption of the immune homeostatic function of lymphatic endothelial cells (LECs) can drive TLS development.

In our ongoing investigation of noncanonical NF-κB signaling in immune homeostasis, we crossed *Ikkα^F/F^* and *Lyve1-cre* mice to create *Ikkα^Lyve-1^* mice that lack IκB kinase (IKK)α in LECs throughout development (21). We previously reported that LN formation is severely impaired in *Ikkα^Lyve-1^* mice (21), and, remarkably, we show in the present study that nonpathogenic BALT spontaneously develops in these mice in the absence of inflammation or tissue damage. Furthermore, following influenza A virus (IAV) infection, *Ikkα^Lyve-1^* mice clear virus and survive despite lacking draining LNs. The lungs of the mice exhibit modestly reduced influenza-induced damage, IFN-γ levels are significantly decreased, and infiltration of CD8 T cells and monocytes is diminished. Thus, we conclude that loss of IKKα signaling in LECs promotes the formation of BALT and reduces proinflammatory stimuli during IAV infection to support improved survival against respiratory virus infection.

## Materials and Methods

### Mice

The *Ikkα^Lyve-1^* mice were described previously by our laboratory (21) and were maintained by breeding *Ikkα^Lyve-1^* mice with *Ikkα^F/F^* mice. For all experiments, *Ikkα^F/F^* littermates were used as controls. Mice were housed in a temperature- and humidity-controlled specific pathogen-free environment with a 12-h light/12-h dark cycle. We found no sex-related differences between *Ikkα^Lyve-1^* and *Ikkα^F/F^* mice at homeostasis or during IAV infection; therefore, we used male and female mice in approximately equal ratios for all experiments.

### Cell culture

Madin-Darby canine kidney (MDCK) cells were cultured and expanded in DMEM (Thermo Fisher Scientific, 11965092) containing 7% FBS (Corning, 35-015-CV) and 50 μg/ml gentamicin (Thermo Fisher Scientific, 15750060).

### Influenza virus titration and infection

H1N1/Puerto Rico/8/34 (PR8) IAV was propagated and titrated for infectivity by half-maximal tissue culture-infective dose (TCID_50_) in MDCK cells following a previously described protocol (22). For infection, mice were anesthetized using 3.5% vaporizer isoflurane and administered virus within 30 μl of PBS intranasally. Mice were monitored for survival and weight loss for the duration of each experiment.

### Histology

Euthanized mice were transcardially perfused with 10 ml PBS, and all harvested organs were submerged in 10% formalin overnight at room temperature. Additionally, lungs were inflated with 1 ml of 10% formalin, and the trachea was sutured. Sample processing and H&E staining were performed at the PennVet Comparative Pathology Core.

### Measuring BALT

The numbers and sizes of BALT in lung sections were quantified from eight unique fields imaged at 5× magnification. Two lung sections were sampled for each mouse. Size was determined by hand outlining BALT using QuPath software (23) and then calculating the area in mm^2^.

### Calculation of mean linear intercept

To determine the alveolar mean linear intercept (MLI), a total of 36 measurements were collected from six unique H&E images from each mouse (5× magnification). For each animal, three lung sections were sampled. ImageJ was used to randomly place six lines of 400-µm length across each image in areas lacking BALT. Linear intercepts with alveolar walls were corrected for the number of intersects and summed to determine the total whole intercepts. The MLI was calculated by dividing the 400-µm line length by the total whole intercept.

### Lung damage assessment

A tile scan of a lung lobe from each mouse was collected at 5× magnification, then stitched images were white balanced and slide imperfections or pieces of extraneous tissue were obscured using ImageJ software so that only the tissue of interest was analyzed. All images were rescaled equivalently so that no image exceeded the 4000 × 4000–pixel dimension limit of the software. Information on the lung damage assessment software is provided in the *Software availability* section below.

### Lung harvest and processing for immunofluorescence microscopy

Euthanized mice were transcardially perfused with 10 ml PBS, then lungs were inflated with 1 ml of 3.2% paraformaldehyde (PFA), and the trachea was sutured closed. Lungs were harvested and submerged in 3.2% PFA, fixed for 1 h at room temperature, then washed three times with PBS over 3 h. Separate lung lobes were submerged in a solution of PBS and 30% sucrose (Sigma-Aldrich, S8501-5KG) overnight at 4°C. Lobes were then submerged in a solution of PBS, 15% sucrose, and 50% Tissue-Tek O.C.T. compound (Sakura, 4583) for 2 h at room temperature prior to snap freezing in O.C.T. using an ethanol dry ice bath.

### Immunofluorescence staining

Lung tissue was cut into 8–10-μm-thick sections on a Leica CM1800 cryostat and mounted on charged microscope slides (Globe Scientific, 1358N). Tissues sections were dipped in 100% acetone (Thomas Scientific, C755J97) for 5 min and then stored at −80°C until use. For staining, the slides were thawed at room temperature, then blocked with a solution of PBS and 5% normal horse serum (Abcam, ab7484) for 1 h at room temperature in a humid chamber. The slides were then washed once with wash buffer (PBS, 1% BSA [Sigma-Aldrich, A9647-100G]). Next, tissue sections were stained with Abs, which included B220-AF488 (RA3-6B2, BD Biosciences, 557669), CD3-allophycocyanin (17A2, BD Biosciences, 565643), CD11c-allophycocyanin (N418, Thermo Fisher Scientific, 17-0114-81), CXCL13 (Polyclonal, Thermo Fisher Scientific, PA5-47018), goat IgG-AF647 (Polyclonal, Abcam, ab150135), ER-TR7 (Novus Biologicals, NB100-64932), rat IgG-AF488 (Polyclonal, Thermo Fisher Scientific, A-21208), LYVE-1-eFluor570 (ALY7, Thermo Fisher Scientific, 41-0443-80), CCL21 (Polyclonal, R&D Systems, AF457), VEGFR3 (Polyclonal, R&D Systems, AF743), and GL7-FITC (BD Biosciences, 562080) as indicated in the figure legends. For primary Abs that did not require a secondary Ab, the tissue was incubated with the Ab mix for 1 h at room temperature in the dark in a humid chamber. For primary Abs requiring a secondary Ab, the tissue was incubated with the primary Ab mix overnight at 4°C in the dark in a humid chamber. The tissue was then washed three times with wash buffer for 5 min. Where applicable, the tissue was incubated with secondary Ab for 1 h at room temperature in the dark in a humid chamber, followed by washing three times with wash buffer for 5 min. The primary Abs for CXCL13, CCL21, and VEGFR3 were followed with the secondary Ab goat-IgG-AF647. The primary Abs for ER-TR7 and GL7 were followed with the secondary Ab rat-IgG-AF488. Tissue sections were then incubated with DAPI (BioLegend, 422801) for 10 min and washed once with wash buffer. Finally, slides were left to dry in the dark at room temperature, overlaid with antifade (Thermo Fisher Scientific, P36930), covered with a coverslip (Warner Instruments, 64-0708), and sealed with clear nail polish.

### Preparation of whole-lung single-cell suspensions

To assess immune cell populations in the lung at homeostasis, euthanized mice were transcardially perfused with 10 ml PBS, and then the lung was filled with 1 ml of enzymatic digestion mix of HBSS+ (Thermo Fisher Scientific, 14025092), DNase I at 200 U/ml (Sigma-Aldrich, 10104159001), collagenase IV at 200 U/ml (Thermo Fisher Scientific, 17104019), and dispase at 0.6 U/ml (Thermo Fisher Scientific, 17105-041), and the trachea was immediately sutured closed. The lung was then placed in 1 ml of enzymatic digestion mix and incubated in a water bath at 37°C for 1 h. Immediately after incubation, the digestion mix was neutralized with 4 ml of a cold HBSS− (Thermo Fisher Scientific, 14175079) and 20% FBS (Corning, 35-015-CV) solution. Next, the lobes of the lung were cut from the trachea and manually digested by pipetting the tissue up and down through a 10-ml serological pipette. The dissociated tissue was passed through a 70-μm cell filter, then resuspended in RBC lysis buffer (Quality Biological Inc, 10128-802) for 5 min on ice. The cells were washed, resuspended in a PBS and 2% FBS solution, and passed through a 40-μm cell filter.

To assess the immune cell populations in the lung at day 7 after IAV infection, mice were first injected with 100 μl of anti-CD45-allophycocyanin Ab (30-F11, BD Biosciences, 559664) at a concentration of 0.024 mg/ml in PBS through the tail vein. Then, 3–5 min after the injection, the mice were euthanized, and the lungs were harvested without PBS perfusion and dissociated to a single-cell suspension as described above.

### Flow cytometry

Single-cell suspensions were incubated with Fc block (2.4G2, BD Biosciences, 553141) and cell viability dye Aqua (Thermo Fisher Scientific, L34966A) for 30 min in the dark at 4°C. Cells were washed twice with FACS buffer (PBS, 2% FBS), then suspended in the indicated Ab mixes for 30 min in the dark at 4°C. The Ab staining mix for the gating strategy in Supplemental Fig. 2A included CD45-PerCP (30-F11, BD Biosciences, 561047), MHC II (I-A/I-E)-PE-Cy5 (M5/114.15.2, BioLegend, 107611), CD24-BV605 (M1/69, BioLegend, 101827), NK1.1-allophycocyanin-Cy7 (PK136, BioLegend, 108723), Ly6G-FITC (1A8, BioLegend, 127605), Ly6C-AF700 (HK1.4, BioLegend, 128023), CD11b-PE (M1/70, BD Biosciences, 557397), CD11c-allophycocyanin (N418, Thermo Fisher Scientific, 17-0114-81), CD4-V450 (RM4-5, BD Biosciences, 560470), and CD8-PE-Cy7 (53-6.7, BD Biosciences, 552877). The Ab staining mix for the gating strategy in Supplemental Fig. 3B included CD45-allophycocyanin (30-F11, BD Biosciences, 559664), B220-PE (RA3-6B2, BD Biosciences, 553089), CD11b-PE (M1/70, BD Biosciences, 557397), CD3-PerCP (145-2C11, BD Biosciences, 553067), CD4-V450 (RM4-5, BD Biosciences, 560470), and CD8-PE-Cy7 (53-6.7, BD Biosciences, 552877). The Ab staining mix for the gating strategy in Supplemental Fig. 3C included CD45-allophycocyanin (30-F11, BD Biosciences, 559664), CD3-allophycocyanin (17A2, BD Biosciences, 565643), B220-AF647 (RA3-6B2, BD Biosciences, 557683), NK1.1-allophycocyanin (PK136, BioLegend, 1087709), Ly6G-BV421 (1A8, BioLegend, 127627), Ly6C-AF700 (HK1.4, BioLegend, 128023), CD11b-PE (M1/70, BD Biosciences, 557397), and CD64-PE-Cy7 (X54-5/7.1, BioLegend, 139313). Afterward, cells were washed twice with FACS buffer, then fixed in PBS containing 2% PFA for 30 min in the dark at room temperature. Data were acquired on a BD LSRFortessa and analyzed using FlowJo version 10 software.

### Bronchoalveolar lavage fluid (BALF) collection and cytokine profiling

BALF was collected in a total of 3 ml of PBS by flushing the lungs with 1 ml of PBS three times. Total protein in the BALF was quantified using a Bradford assay kit (Thermo Fisher Scientific, 23236), with each biological sample run in duplicate. Select cytokines in BALF were measured using the MILLIPLEX Mouse Cytokine/Chemokine Magnetic Bead Panel - Premixed 25 Plex multiplex assay (Sigma-Aldrich, MCYTOMAG-70K-PMX). The assay was performed at the University of Pennsylvania Human Immunology Core using the Luminex xMAP platform, and each biological sample was run in duplicate.

### Pulse oximetry

Measurement of capillary oxygen saturation (SpO_2_) was performed using a MouseOx Plus Rat & Mouse Pulse Oximeter and a MouseOx small collar sensor (Starr Life Sciences Corp.). Measurements were recorded using MouseOx Premium Software (Starr Life Sciences Corp). At least 1 d prior to taking the first measurements, mice were shaved around the neck to allow the collar to contact skin. Readings were taken for >3 min continuously at a rate of 15 Hz, and the mice were awake for the entirety of the measurements. The measurements were then assessed in Excel for quality, and any readings that were associated with an error, except for lost breath Rate, were excluded from analysis. The remaining readings were averaged to calculate SpO_2_%.

### Lung viral load

To collect IAV-infected lung supernatants, mice were euthanized on day 6, 7, 8, or 9 postinfection. Lungs were harvested and placed in 1 ml solution of MEM with no glutamine (Thermo Fisher Scientific, 11090081) with GlutaMAX (1×) (Thermo Fisher Scientific, 35050061) and gentamicin at 50 μg/ml. Next, lungs were pulverized for 2 min, centrifuged at 1800 rpm for 8 min, and the supernatant was collected. The samples of supernatant were snap frozen in an ethanol and dry ice bath and stored at −80°C until use.

To titer lung supernatants, 1 d prior to the assay, flat-bottomed 96-well plates were seeded with MDCK cells at 4 × 10^4^ cells/well. The next day the wells were 90–100% confluent. Cells were washed twice with MEM (no serum) and then 180 μl of TCID_50_ media (MEM with GlutaMAX [1×], 50 μg/ml gentamicin, and 1 μg/ml L-1-tosylamido-2-phenylethyl chloromethyl ketone–treated trypsin [Worthington Biochemical, LS003740]) was added to each well. The lung supernatant samples were thawed in a 37°C water bath, then centrifuged at 8000 rpm for 5 min. A quantity of 20 μl of sample was added in quadruplicate to the first well of the plate, then 11 serial dilutions were made, and the wells in row 12 served as no-virus controls. The plate was incubated for 4 d at 37°C/5% CO_2_, then wells positive for IAV were read for cytopathic effect using a light microscope. Wells with pathology were recorded, and the TCID_50_/ml viral load was calculated using the Reed and Muench calculation.

### Imaging

All images, single or tile scans, were obtained using a Leica DM6000B upright widefield EL6000 fluorescence light microscope fitted with a Leica DMC-2900 and Hamamatsu Orca 03G charge-coupled device camera using LAS X software. Images were handled in ImageJ software, and processing was minimal and included white balancing for H&E images and/or slight adjustment of contrast and brightness for IF microscopy images.

### Statistics

Statistical analyses were performed using GraphPad Prism version 10 software. Comparisons between two groups were made using a two-tailed unpaired *t* test, and when two sets of data did not have equal variances, a Welch correction was applied. Normality of data was determined using the Shapiro-Wilk test and the Kolmogorov-Smirnov test. Differences in survival were analyzed by Kaplan-Meier survival analysis. Values of *p *<* *0.05 were considered significant. Data are presented as mean ± SD as designated in the figure legends.

### Study approval

All animal studies were approved by the University of Pennsylvania Institutional Animal Care and Use Committee and in accordance with the NIH Office of Laboratory Animal Welfare regulations.

### Software availability

QuPath is open-source software (23). Lung damage assessment software (LungDamage) is publicly available on Github (https://github.com/WALIII/LungDamage) (24).

## Results

### The lungs of Ikkα^Lyve-1^ mice contain distinct immune cell clusters in the absence of inflammation

**FIGURE 1.**
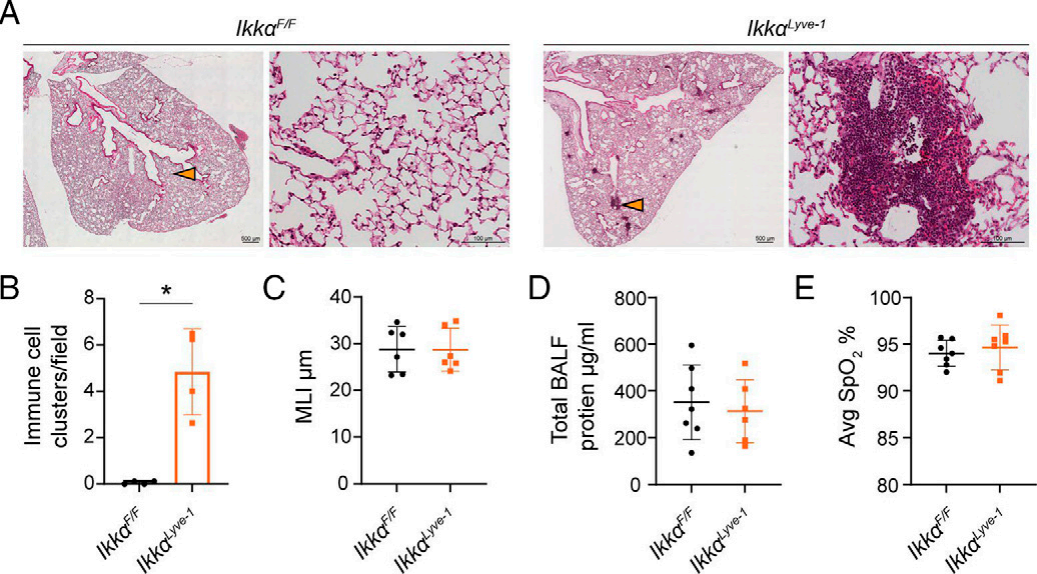
Discrete clusters of immune cells are present in the lungs of *Ikkα^Lyve-1^* mice in the absence of inflammation. (**A**) Representative H&E-stained, paraffin-embedded lung sections from *Ikkα^F/F^* and *Ikkα^Lyve-1^* mice. For each mouse, lefthand images are tile scans of a lung lobe (scale bars, 500 μm), and righthand images are a single image of the tissue indicated by the orange arrow (scale bars, 100 μm). (**B**) Average number of immune cell clusters within a field of view (5× magnification). Mice were 13 wk of age; *n =* 4 mice. Statistics were calculated by unpaired *t* test with Welch correction. *p <* *0.05. Data represent the mean ± SD. (**C**) Quantification of alveolar morphology by MLI. Mice were 13 wk of age; *n =* 6 mice. (**D**) Measurement of total protein in BALF by Bradford assay. Mice were 10 wk of age; *n =* 6–7 mice. (**E**) Pulse oximetry readings of SpO_2_%. Mice were 10 wk of age; *n =* 7 mice. (C–E) Statistics were calculated by unpaired *t* test. Data represent the mean ± SD.

We found previously that *Ikkα^Lyve-1^* mice lack all peripheral LNs (21); however, we did not determine if loss of LEC-intrinsic IKKα leads to pathophysiological defects in nonlymphoid tissues. To address this, we performed comprehensive histological evaluation of tissues, including the lungs, liver, kidney, pancreas, intestine, and mesentery. Strikingly, although all other tissues examined appeared normal (Supplemental Fig. 1A), the lungs of *Ikkα^Lyve-1^* mice contained discrete immune cell clusters adjacent to blood vessels and along the bronchial tree (Fig. 1A). Quantitation of these aggregates revealed that they occur significantly more frequently in *Ikkα^Lyve-1^* mice than in *Ikkα^F/F^* littermate controls, in which immune cells were sparse and clusters were rarely observed (Fig. 1A, 1B).

Immune cell infiltration into nonlymphoid tissues is a hallmark of inflammation; therefore, we assessed the lungs of *Ikkα^Lyve-1^* mice for evidence of inflammation and associated damage. Because inflammation-induced lung injury can lead to enlargement and loss of alveolar morphology, we quantitated alveolar size by MLI analysis in lung sections from *Ikkα^F/F^* and *Ikkα^Lyve-1^* mice. As shown in Fig. 1C, no significant differences were detected in the MLI of *Ikkα^Lyve-1^* lungs compared with controls. We next determined the concentration of total protein (a surrogate for impaired vascular barrier function) in the BALF and again found no difference between *Ikkα^Lyve-1^* mice and *Ikkα^F/F^* littermate controls (Fig. 1D). Finally, because overall lung function is sensitive to injury, we measured the SpO_2_ in conscious mice, and, consistent with our other analyses, no significant differences were detected in SpO_2_% between *Ikkα^Lyve-1^* and *Ikkα^F/F^* control mice (Fig. 1E).

Next, we asked if the clusters arise as a prenatal developmental defect in *Ikkα^Lyve-1^* mice or if they appear after birth. Evaluation of lung sections from 1-, 3-, 6-, 13-, 26-, and 52-wk-old *Ikkα^Lyve-1^* mice revealed that aggregates are not present until ∼3 wk of age, after which they increase in number and size until ∼26 wk (Supplemental Fig. 1B, 1C). Taken together, these findings reveal that clusters of immune cells spontaneously arise postnatally in the lungs of *Ikkα^Lyve-1^* mice in the absence of inflammation or apparent damage.

### The immune cell aggregates in Ikkα^Lyve-1^ lungs are organized ectopic lymphoid structures

**FIGURE 2.**
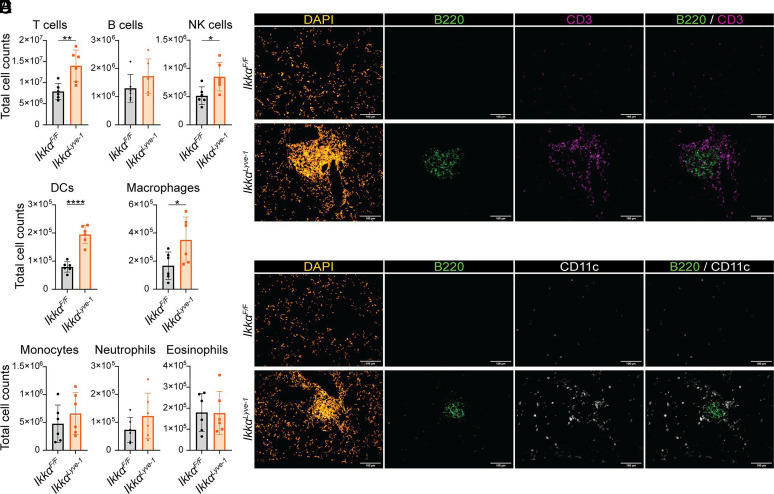
The immune cell aggregates contain organized B and T cell zones and CD11c^+^ DCs. (**A–C**) Flow cytometry of immune cells at homeostasis from perfused, homogenized lungs. See Supplemental Fig. 2A for gating strategy (22). Mice were 10 wk of age; *n =* 6 mice. Statistics were calculated by unpaired *t* test. *p <* *0.05; **p <* *0.01; ***p <* *0.001; ****p <* *0.0001. Data represent the mean ± SD. (**D**) Staining for B cells (B220, green) and T cells (CD3, purple) in the lung. (**E**) Staining for B cells (B220, green) and DCs (CD11c, white) in the lung. (D and E) DAPI (yellow) was used to stain cell nuclei. Scale bars, 100 μm; mice were 10 wk of age.

To identify the immune cell populations present in the lungs of *Ikkα^Lyve-1^* mice at homeostasis, we performed comprehensive flow cytometry (25) on perfused lung homogenates (Supplemental Fig. 2A). Consistent with the presence of immune cell clusters in *Ikkα^Lyve-1^* lungs, total CD45^+^ cells were significantly increased compared with *Ikkα^F/F^* littermate controls (Supplemental Fig. 2B). Most immune cells in both *Ikkα^Lyve-1^* and *Ikkα^F/F^* lungs were lymphocytes, including CD4^+^ T cells, CD8^+^ T cells, and NK cells, and the numbers of each of these subsets were significantly elevated in *Ikkα^Lyve-1^* lungs (Fig. 2A and Supplemental Fig. 2B). Although B cells were not significantly different between the groups, B cell numbers trended toward an increase in *Ikkα^Lyve-1^* lungs (Fig. 2A). Macrophages and DC populations were also significantly increased in the lungs of *Ikkα^Lyve-1^* mice (Fig. 2B), whereas the numbers of other innate immune cells (monocytes, neutrophils, and eosinophils) were no different between *Ikkα^Lyve-1^* and *Ikkα^F/F^* lungs (Fig. 2C). Together these data reveal that lymphocyte and APC populations are significantly increased in the lungs of *Ikkα^Lyve-1^* mice.

To determine if these elevated immune cell populations are associated with the leukocyte clusters in *Ikkα^Lyve-1^* lungs (Fig. 1), we investigated their location and organization by immunofluorescence (IF) microscopy of frozen lung sections. As shown in Fig. 2D, B220^+^ B cells and CD3^+^ T cells were sparsely distributed throughout the lung parenchyma of *Ikkα^F/F^* control mice. In contrast, B and T cells in *Ikkα^Lyve-1^* lungs were immediately apparent as accumulated populations forming the immune cell clusters (Fig. 2D). Moreover, these lymphocytes were arranged into distinct zones, with B cells concentrated as a central follicle largely surrounded by the T cell population. CD11c^+^ DCs were also present within the clusters, and these were predominantly localized at the edge of the B cell zone (Fig. 2E). These findings therefore identify the aggregates in the lungs of *Ikkα^Lyve-1^* mice as organized ectopic lymphoid structures consisting of distinct zones of B and T lymphocytes and APCs.

### The lymphoid structures in Ikkα^Lyve-1^ mice are mature BALT

The organized nature of the lymphoid aggregates in the lungs of *Ikkα^Lyve-1^* mice resembles disease-associated iBALT (6). In addition to containing organized immune cell compartments, iBALT, like TLSs, also exhibits reorganized stromal cells, lymphatics, and blood vasculature distinct from the surrounding parenchyma (4). We therefore performed IF microscopy to determine if the ectopic lymphoid structures in *Ikkα^Lyve-1^* mice display these characteristics of mature TLSs.

**FIGURE 3.**
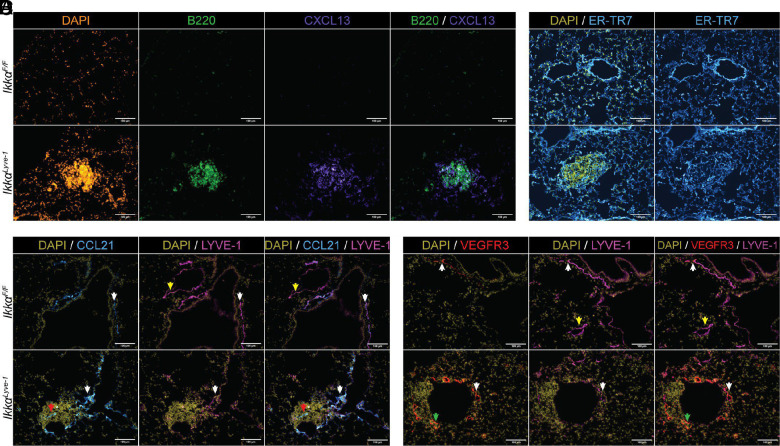
The lymphoid structures in *Ikkα^Lyve-1^* lungs are BALT. (**A**) Staining for DAPI (orange), B cells (B220, green), and CXCL13 (purple) in the lung. (**B**) Staining for DAPI (yellow) and ER-TR7 (blue). (**C**) Staining for DAPI (yellow), CCL21 (blue), and LYVE-1 (purple). White arrows indicate a vessel that is positive for CCL21 and LYVE-1, yellow arrows indicate a vessel that is positive for LYVE-1 and negative for CCL21, and red arrows indicate a vessel that is positive for CCL21 and negative for LYVE-1. (**D**) Staining for DAPI (yellow), VEGFR3 (red), and LYVE-1 (purple). White arrows indicate a vessel that is positive for VEGFR3 and LYVE-1, yellow arrows indicate a vessel that is positive for LYVE-1 and negative for VEGFR3, and green arrows indicate a vessel that is positive for VEGFR3 and negative for LYVE-1. (A–D) Scale bars, 100 μm; mice were 10 wk of age.

The stroma of TLSs must reorganize to support lymphoid follicle formation. CXCL13 is expressed on the surface of follicular DCs that form the stroma of B cell follicles in secondary lymphoid organs (1) and has been shown to be induced in TLSs, including iBALT (6). CXCL13 was not detected in the lungs of control *Ikkα^F/F^* mice; however, it was robustly associated with the lymphoid structures in *Ikkα^Lyve-1^* lungs (Fig. 3A). The strongest CXCL13 expression occurred in the area occupied by B220^+^ B cells and was also detected in regions immediately surrounding the B cell follicle (Fig. 3A). Notably, CXCL13 was restricted to only the lymphoid structures and was not detected in any other areas of the lungs in *Ikkα^Lyve-1^* mice. To evaluate the overall organization of stromal cells, we stained lung sections with the Ab ER-TR7 that specifically recognizes collagen VI on fibroblasts (26). Although ER-TR7 staining was abundant throughout normal lung tissue in both *Ikkα^Lyve-1^* and *Ikkα^F/F^* mice, the organization of fibroblastic cells at the site of the lymphoid follicles in *Ikkα^Lyve-1^* lungs was visibly distinct, because the ER-TR7^+^ network was substantially denser than the surrounding tissue (Fig. 3B). Taken together, our data indicate that *Ikkα^Lyve-1^* lungs contain distinct areas of remodeled stromal cells that can support and maintain lymphoid follicles in TLSs.

Previous studies have established that iBALT occurs proximal to lymphatic vessels (2). To assess the lymphatic environment associated with the lymphoid structures in *Ikkα^Lyve-1^* mice, we performed IF analysis using Abs against the lymphatic markers CCL21, LYVE-1, and VEGFR3. Lymphatics were identified in *Ikkα^F/F^* control and *Ikkα^Lyve-1^* lungs by dual expression of CCL21 and LYVE-1 (Fig. 3C) or expression of VEGFR3 (Fig. 3D). In *Ikkα^Lyve-1^* mice, we observed CCL21^+^; LYVE-1^+^ (Fig. 3C) and VEGFR3^+^; LYVE-1^+^ (Fig. 3D) lymphatic vessels adjacent to the lymphoid structures (white arrows in each figure). In addition, VEGFR3^+^; LYVE-1^−^ vessels were present on the edges of the aggregates (Fig. 3D, green arrow). It is likely that these are lymphatic collecting vessels that have markedly reduced LYVE-1 expression in comparison with capillary lymphatics (27). These findings therefore reveal that an extensive lymphatic network consisting of capillary and collecting vessels surrounds the lymphoid structures in *Ikkα^Lyve-1^* lungs.

In addition to lymphatics, we also observed LYVE-1 staining of vessels that did not express either CCL21 or VEGFR3 in both *Ikkα^F/F^* control and *Ikkα^Lyve-1^* mice. These vessels, as indicated by yellow arrows in the *Ikkα^F/F^* images in Fig. 3C, 3D, are most likely LYVE-1^+^ blood vessels that have previously been described in the lungs (19, 28). Finally, we sought to determine if the BALT structures contained HEVs, which are considered a hallmark of tertiary lymphoid tissues (1). Unlike all other blood vessels, HEVs homeostatically express CCL21 together with specific adhesion molecules, including peripheral node addressin (PNAd), which can be detected using the Ab MECA-79. Remarkably, although MECA-79 has been shown previously to identify HEVs in disease-associated iBALT (6), we did not detect MECA-79^+^ vessels in *Ikkα^Lyve-1^* lungs. Intriguingly, however, we identified CCL21^+^; LYVE-1^−^ structures within the lymphoid aggregates, consistent with the previously described location of HEVs in iBALT (6) (Fig. 3C, red arrows). Our accumulated findings lead us to conclude that the lymphoid aggregates present in *Ikkα^Lyve-1^* mice are novel mature BALT structures that are distinct from iBALT because they develop in the absence of inflammation or any disease-associated pathology.

### Ikkα^Lyve-1^ mice exhibit reduced morbidity and mortality following influenza infection

**FIGURE 4.**
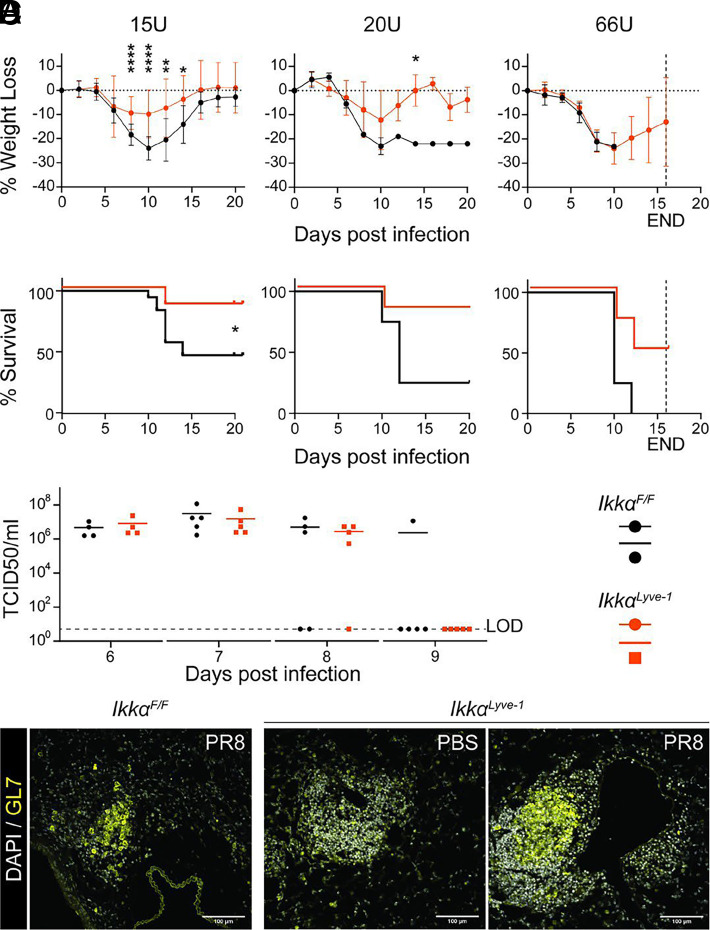
*Ikkα^Lyve-1^* mice display improved survival following influenza infection. (**A**) Percent weight loss over the course of infection. Statistics were calculated by unpaired *t* test *p <* *0.05; **p <* *0.01; ****p <* *0.0001. Data represent the mean ± SD. (**B**) Percent survival over the course of infection. Statistics were calculated by Kaplan-Meier survival analysis. *p <* *0.05. (A and B) *Ikkα^F/F^* (black lines and points) and *Ikkα^Lyve-1^* mice (orange lines and points) were infected with 15U, 20U, or 66U TCID_50_ units of PR8 virus. All mice were 10–12 wk of age. END, end of study. At 15U, *n =* 15–19 mice; at 20U, *n =* 4–6 mice; and at 66U, *n =* 4 mice. (**C**) Viral load from supernatants of dissociated lungs from *Ikkα^F/F^* (black points) and *Ikkα^Lyve-1^* mice (orange points). Mice were infected with 15U TCID_50_ units of PR8 virus. LOD, limit of detection. Mice were 8–10 wk of age; *n =* 4–5 mice. Data represent the mean. (**D**) Staining for DAPI (white) and GL7 (yellow) in lung tissue sections 10 d after treatment with PBS or 15U TCID_50_ units of PR8. Scale bars, 100 μm.

Earlier studies established that infection with respiratory viruses drives the formation of iBALT, which participates in local antiviral immune responses (6, 7, 9). To determine if the preexisting BALT in *Ikkα^Lyve-1^* mice plays a protective or pathogenic role in response to respiratory virus infection, we challenged *Ikkα^Lyve-1^* and *Ikkα^F/F^* littermate control mice with increasing doses of PR8 IAV and monitored weight loss and survival for up to 20 d postinfection. As shown in Fig. 4A, 4B, *Ikkα^F/F^* control mice lost weight and displayed significant mortality when challenged with 15 TCID_50_ units of PR8. In contrast *Ikkα^Lyve-1^* mice did not lose significant weight, and almost all mice survived this dose of virus. Furthermore, when challenged with increasingly lethal doses of PR8, *Ikkα^Lyve-1^* mice consistently exhibited reduced morbidity and mortality compared with *Ikkα^F/F^* controls, indicating less susceptibility to IAV infection (Fig. 4A, 4B).

To determine if the improved morbidity and mortality of *Ikkα^Lyve-1^* mice was associated with a more robust antiviral response, we evaluated the viral load in the lungs of *Ikkα^F/F^* and *Ikkα^Lyve-1^* mice following IAV infection. Strikingly, the viral load and the rate of viral clearance were no different between the groups of mice (Fig. 4C), indicating that *Ikkα^F/F^* and *Ikkα^Lyve-1^* mice mount equally effective immune responses to IAV. Because *Ikkα^Lyve-1^* mice lack all LNs, these findings suggest that the preexisting BALT participates in the immune response to IAV. To evaluate this, we performed IF microscopy using anti-GL7 to detect GC cells. At day 10 after PR8 challenge, GC cells were present in the lungs of control *Ikkα^F/F^* mice (Fig. 4D), where they appeared as small, loose clusters consistent with the previously described induction of iBALT (6). In *Ikkα^Lyve-1^* mice treated with PBS, preexisting BALT did not contain GC cells, providing further support that the structures develop in the absence of inflammatory or immune stimuli (Fig. 4D). However, at 10 d post-challenge, large GCs were present in *Ikkα^Lyve-1^* mice, revealing that the BALT actively responds during the antiviral immune response (Fig. 4D). Together these data indicate that preexisting BALT in *Ikkα^Lyve-1^* mice is sufficient to provide an antiviral response; however, these results do not reveal why *Ikkα^Lyve-1^* mice have a significant survival advantage over *Ikkα^F/F^* littermates, because viral replication and spread is controlled equally by both groups.

### IFN-γ and infiltrating CD8 T cells and monocytes are significantly decreased in Ikkα^Lyve-1^ lungs during influenza infection

**FIGURE 5.**
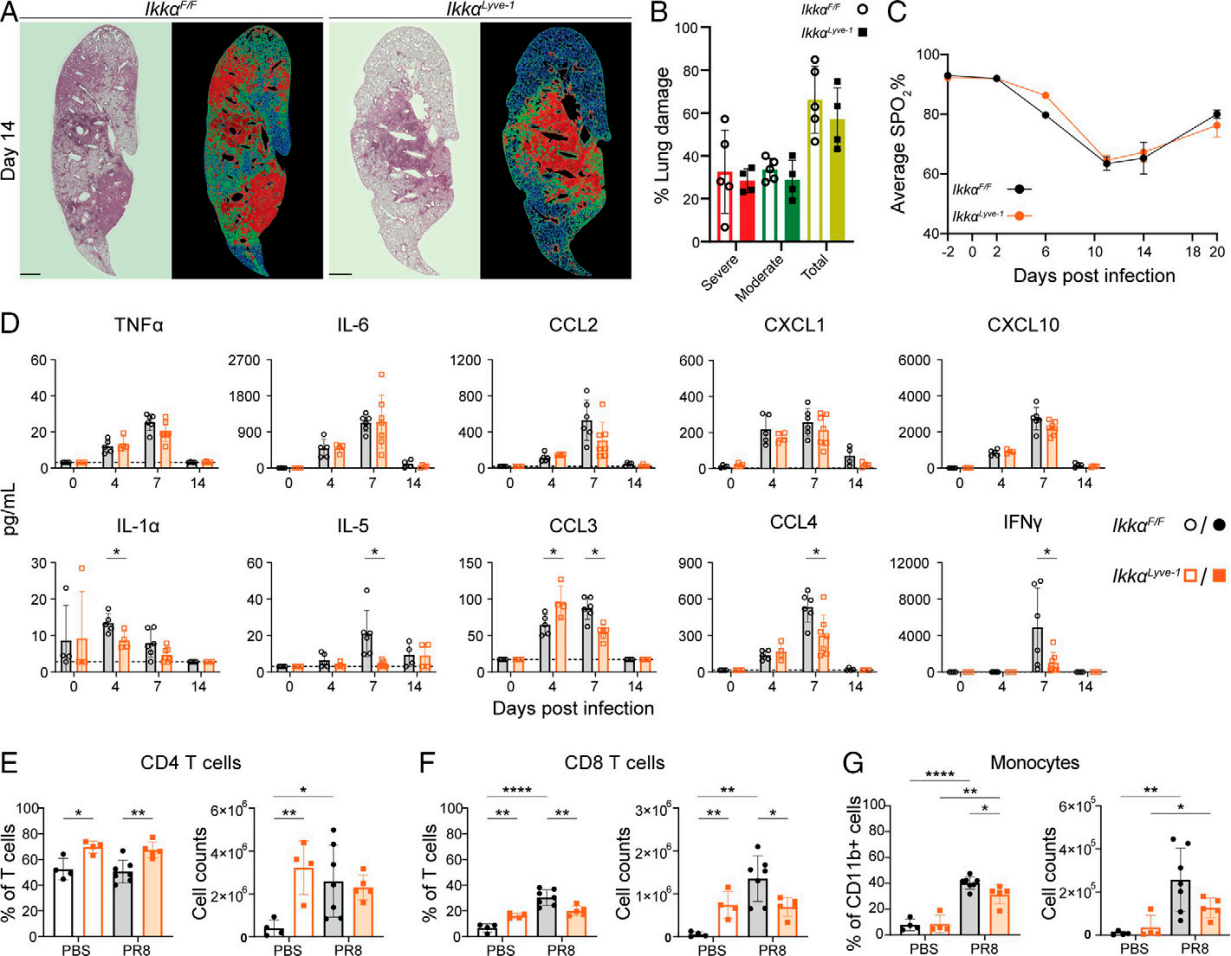
*Ikkα^Lyve-1^* mice have reduced levels of IFN-γ and CD8 T cells and monocytes in the lung during influenza infection. (**A**) Representative images of H&E-stained, paraffin-embedded lung lobe sections and the corresponding analyzed image from day 14 after influenza infection. The image is a tile scan of a lung lobe. Severe damage (red), moderate damage (green), and normal lung (blue). Scale bars, 1000 μm. (**B**) Percentage of lung damage by damage type. Total damage is the sum of severe and moderate damage. (A and B) *n =* 4–5 mice. (**C**) Pulse oximetry readings of SpO_2_% throughout infection. *n =* 8–13 mice. (**D**) Level of cytokines and chemokines in BALF throughout influenza infection. *n =* 4–7 mice. (**E**) Flow cytometry for intralung CD4 T cells. (**F**) Flow cytometry for intralung CD8 T cells. (**G**) Flow cytometry for intralung monocytes. (E–G) Immune cell populations in the lung from day 7 after influenza infection. See Supplemental Fig. 3B, 3C, for gating strategy. *n =* 4–7 mice. (A–G) Mice were infected with 15U TCID_50_ units of PR8. Mice were 10–11 wk of age. Statistics were calculated by unpaired *t* test *p <* *0.05; **p <* *0.01; ***p <* *0.001; ****p <* *0.0001. Data represent the mean ± SD.

Influenza infection leads to extensive inflammation and associated lung tissue damage, resulting in the disruption of lung function. Because *Ikkα^Lyve-1^* mice clear PR8 at the same rate as *Ikkα^F/F^* littermates but exhibit significantly enhanced survival (Fig. 4B, 4C), we asked if differences in IAV-induced lung injury exist between the mice. To assess injury, groups of *Ikkα^F/F^* and *Ikkα^Lyve-1^* mice were infected with PR8, then lungs were excised at day 14 postinfection, when damage is maximal. As expected, H&E staining of lung sections from *Ikkα^F/F^* mice revealed regions of fibrotic scarring and loss of alveolar integrity, indicating widespread lung injury from influenza infection (Fig. 5A, left panels). Substantial areas of injured tissue were also present in *Ikkα^Lyve-1^* lungs; however, larger regions of intact lung parenchyma were also visible when compared with *Ikkα^F/F^* littermates. To quantify tissue injury, we employed a novel unbiased computational image analysis platform to identify moderate, severe, and total damage on the basis of intensity of H&E staining (Fig. 5A, right panels) (24). As shown in Fig. 5B, no injury category was significantly different between *Ikkα^F/F^* and *Ikkα^Lyve-1^* mice; however, the mean percentage damage in each classification was lower in *Ikkα^Lyve-1^* mice. We next evaluated lung function in groups of *Ikkα^F/F^* and *Ikkα^Lyve-1^* mice by measuring SpO_2_ through the course of IAV infection and recovery. As shown in Fig. 5C, the decrease and rebound of SpO_2_% during infection and clearance was virtually identical between *Ikkα^F/F^* and *Ikkα^Lyve-1^* mice. Taken together, these data reveal that although IAV infection causes marginally less tissue injury in *Ikkα^Lyve-1^* mice, the reduced morbidity and mortality are not due to a significant impact on pathology or lung function.

To determine if the improved survival of *Ikkα^Lyve-1^* mice is associated with altered or diminished inflammation, we performed multiplex analysis of 25 proinflammatory cytokines and chemokines in BALF from *Ikkα^F/F^* and *Ikkα^Lyve-1^* mice at days 0 (uninfected mice), 4, 7, and 14 after IAV infection. Nine analytes were not detected (Supplemental Table I), and 10 analytes that were detected were not significantly different between *Ikkα^F/F^* and *Ikkα^Lyve-1^* mice at any time point (Supplemental Table I). Of these 10 analytes, TNF-α, IL-6, CCL2, CXCL1, and CXCL10 have been shown previously to play major roles in IAV-induced inflammation (29). Each of these analytes was robustly increased in BALF at days 4 and 7 but were not different between *Ikkα^F/F^* and *Ikkα^Lyve-1^* mice (Fig. 5D); therefore, the improved survival of *Ikkα^Lyve-1^* mice is not due to changes in expression of these major drivers of inflammation. Notably, however, six analytes displayed significant differences between *Ikkα^Lyve-1^* and *Ikkα*^F/F^ control mice, and, of these, G-CSF and CCL3 (MIP-1α) were increased in *Ikkα^Lyve-1^* mice at day 4 postinfection (Supplemental Fig. 3A and Fig. 5D). In contrast, IL-1α was decreased at day 4 after infection, whereas IL-5, CCL3, CCL4 (MIP-1β), and IFN-γ were significantly reduced in *Ikkα^Lyve-1^* BALF at day 7 after infection (Fig. 5D). Thus, a subset of inflammatory mediators that recruit, activate, and modulate both innate and adaptive immune responses are reduced in the lungs of *Ikkα^Lyve-1^* mice.

The significant decrease in IFN-γ in *Ikkα^Lyve-1^* BALF (Fig. 5D) was particularly intriguing because loss of either IFN-γ or the IFN-γ receptor or the administration of IFN-γ neutralizing Abs reduces IAV-associated inflammation and improves survival (30–32). Because CD8 T cells are the major producers of IFN-γ during the immune response to influenza (33), we analyzed T cell populations in the lungs of PR8-challenged *Ikkα^F/F^* and *Ikkα^Lyve-1^* mice at 7 d after infection. Immune cell infiltrates were distinguished from circulating cells in the lung through i.v. injection of anti-CD45 Ab prior to euthanasia. As shown in Fig. 5E, 5F, when comparing the PBS-treated groups, CD4 and CD8 T cell numbers were significantly elevated in *Ikkα^Lyve-^*^1^ lungs, a finding consistent with the increased numbers of T cells in the lungs at homeostasis (Fig. 2). During IAV infection, both CD4 and CD8 T cell numbers increased in *Ikkα^F/F^* mice (Fig. 5E, 5F). Remarkably, in IAV-infected *Ikkα^Lyve-^*^1^ lungs, T cell numbers did not increase beyond levels in PBS-treated *Ikkα^Lyve-^*^1^ lungs. Moreover, although the numbers of CD4 T cells in IAV-infected *Ikkα^F/F^* and *Ikkα^Lyve-^*^1^ mice were similar between both groups at day 7 after infection (Fig. 5E), CD8 T cell numbers and frequency were significantly lower in *Ikkα^Lyve-1^* lungs (Fig. 5F). These data show that infection with IAV does not cause a significant increase in the number of T cells in the lungs of *Ikkα^Lyve-1^* mice and that the numbers and frequency CD8 T cells are significantly lower than those in IAV-infected *Ikkα^F/F^* mice. Collectively, these findings indicate that infiltration of T lymphocytes into the lungs following IAV infection is severely attenuated in *Ikkα^Lyve-1^* mice.

**FIGURE 6.**
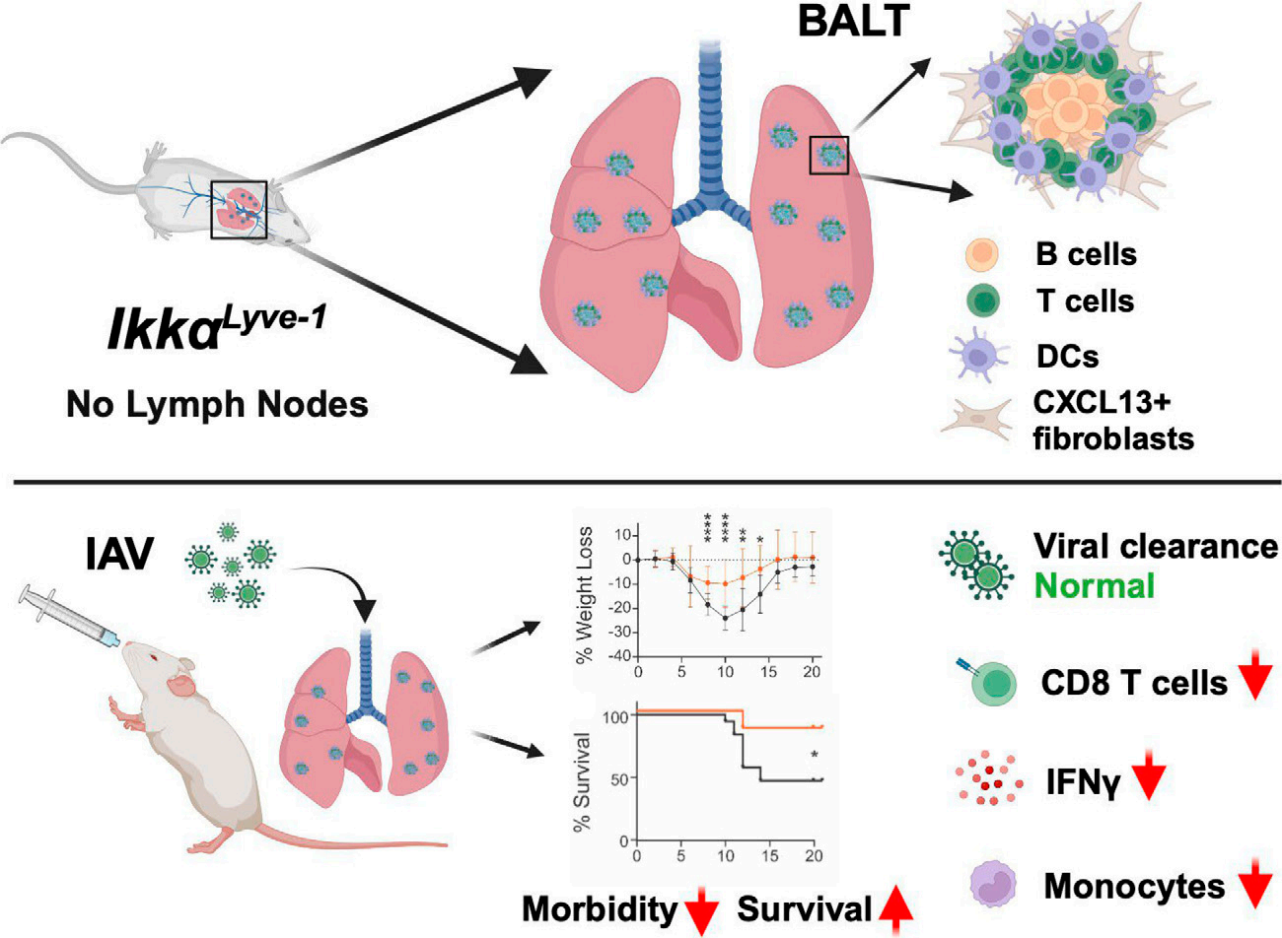
*Ikkα^Lyve-1^* mice develop nonpathogenic BALT and are protected from IAV infection. *Ikkα^Lyve-1^* mice have no LNs (21), but we now show that they spontaneously develop organized BALT in the absence of inflammation or damage. Following IAV infection, *Ikkα^Lyve-1^* mice exhibit improved morbidity and survival (data taken from Fig. 4; *Ikkα^F/F^*, black lines; *Ikkα^Lyve-1^*, orange lines), and, despite lacking all LNs, they clear virus as efficiently as *Ikkα^F/F^* littermate controls. Levels of IFN-γ and the numbers of CD8 T cells and monocytes are substantially decreased in IAV-infected *Ikkα^Lyve-1^* lungs compared with *Ikkα^F/F^* littermates, indicating that improved survival results from reduced inflammatory stimuli. Figure created with Biorender.com.

We also investigated innate immune cell populations and found that the numbers of neutrophils and macrophages were increased to similar levels in the lungs of *Ikkα^F/F^* and *Ikkα^Lyve-1^* mice at 7 d following PR8 infection (Supplemental Fig. 3D). Importantly, it was shown recently that IFN-γ drives inflammation and morbidity during IAV infection via the recruitment of monocytes into the lungs (33). We therefore compared the frequency and numbers of monocytes in the lungs of PBS-treated and PR8-challenged *Ikkα^F/F^* and *Ikkα^Lyve-1^* mice. As shown in Fig. 5G, monocytes were infrequent in PBS-treated *Ikkα^F/F^* and *Ikkα^Lyve-1^* mice, and there were no differences in their numbers between these groups. Following IAV infection, monocyte numbers in both mice were increased; however, this infiltration was substantially reduced in *Ikkα^Lyve-1^* mice compared with *Ikkα^F/F^* littermate controls (Fig. 5G). Taken together, our findings reveal that IFN-γ and a subset of proinflammatory cytokines are significantly decreased in the lungs of PR8-infected *Ikkα^Lyve-1^* mice. This is accompanied by lower numbers of CD8 T cells and monocytes, leading us to conclude that *Ikkα^Lyve-1^* mice survive lethal influenza infection through reduced recruitment of specific proinflammatory immune cells into the lungs (Fig. 6).

## Discussion

We reported previously that *Ikkα^Lyve-1^* mice lack all peripheral LNs, supporting a central function for LEC-intrinsic IKKα in the normal formation of these organs (21). We now show that *Ikkα^Lyve-1^* mice develop BALT in their lungs that display the hallmarks of TLSs, including compartmentalized B and T cell/DC zones, reorganized stroma, and blood and lymphatic vessel networks (Fig. 6). Extensive studies have described the formation and function of iBALT in a wide range of diseases, including infection with respiratory pathogens, chronic obstructive pulmonary disease, and asthma (3, 4), and, in each of these conditions, iBALT forms in response to inflammation that drives immune cell infiltration and tissue injury. In marked contrast to disease-associated iBALT, the BALT in *Ikkα^Lyve-1^* mice develops spontaneously in the absence of inflammation or lung damage. Consequently, our findings indicate that loss of IKKα in LECs disrupts normal lung immune homeostasis and results in nonpathogenic BALT formation.

The role of TLSs in immune diseases and cancer is an area of intense interest because, depending on the context, TLSs either exacerbate pathology or play beneficial roles in disease resolution (1, 5). Significant effort focuses on understanding the cells and signals that control TLS formation and function; however, the role of LECs is poorly defined (2). Intriguingly, like *Ikkα^Lyve-1^* mice, *Clec2^−/−^* mice develop BALT, and TLSs do not form in any other tissues (19). Importantly, forward lymph flow and leukocyte egress via lung lymphatics are severely impaired in *Clec2^−/−^* mice, suggesting that BALT is induced because immune cell trafficking from the lung is impaired (19, 20). BALT also develops in mice lacking CCR7 on CD11c^+^ DCs (34–36) because CCR7^−/−^ DCs cannot recognize LEC-expressed CCL21, thus preventing their exit from the lung (36). DCs are crucial for forming and maintaining iBALT in IAV-infected mice (8, 37), but CCR7^−/−^ DCs were able to induce BALT formation in the absence of inflammation, similar to *Ikkα^Lyve-1^* mice. We find accumulation of DCs together with lymphocytes in *Ikkα^Lyve-1^* mice, suggesting that their normal egress is similarly impaired. However, because *Ikkα^Lyve-1^* mice lack LNs (21), we cannot directly evaluate immune cell migration via the draining LNs, but together with these prior studies, our data suggest that loss of LEC-intrinsic IKKα disrupts normal lymphatic trafficking of immune cells, leading to BALT formation.

We employed the widely used constitutive *Lyve1-cre* model to investigate how the loss of IKKα in LECs affects lymphoid organogenesis (38). However, LYVE-1 is not fully restricted to LECs and is expressed by some blood endothelial cells (BECs) and a subset of tissue-resident interstitial LYVE-1^+^ macrophages (39). In the lung, LYVE-1 is expressed by embryonic blood capillaries (40), and, consistent with previous work (19, 28), we observed some LYVE-1^+^ blood vessels in both *Ikkα^F/F^* and *Ikkα^Lyve-1^* lungs. Because IKKα will be deleted in these vessels in *Ikkα^Lyve-1^* mice, this may play a role in BALT formation. However, our previous work and that of others (41, 42) suggest that in limited scenarios, noncanonical NF-κB signaling in BECs can support lymphocyte migration, and, if such regulation exists for homeostatic leukocyte entry into the lung, this would be inhibited in *Ikkα^Lyve-1^* mice. Because we find increased numbers of immune cells, including lymphocytes and DCs, in *Ikkα^Lyve-1^* lungs, our data indicate that loss of IKKα function in BECs does not impede leukocyte entry.

IKKα will also be deleted in LYVE-1^+^ macrophages in *Ikkα^Lyve-1^* mice, but no role for IKKα signaling has been defined in this population. Notably, the major function of lung-resident LYVE-1^+^ macrophages is maintenance of blood vessel integrity, and loss of these cells drives inflammation and vascular leak (39). Because BALT forms in the *Ikkα^Lyve-1^* mice without inflammation or discernible lung damage, this role of LYVE-1^+^ macrophages appears unaffected by the loss of IKKα. Thus, although we cannot fully discount a role for LYVE-1^+^ BECs or macrophages in BALT formation and further study of how IKKα regulates these cell types is required, their contribution, if any, is not related to their known functions in normal immune homeostasis. Instead, our findings strongly suggest that within the overall LYVE-1^+^ cellular compartment, loss of IKKα in LECs and the effects of this loss on homeostatic immune cell egress are the major mechanism driving BALT formation.

BALT in *Ikkα^Lyve-1^* mice is remarkably similar to previously described iBALT (4), including the presence of a B follicle associated with CXCL13-expressing stromal cells, T cells, DCs, and unique vasculature. Formation of HEVs, which normally exist only in LNs and Peyer’s patches, is another hallmark of TLSs (2). To identify HEVs, we employed the MECA79 Ab that recognizes a modified epitope of PNAd; however, we found no PNAd^+^ vessels in *Ikkα^Lyve-1^* mice. HEVs also express CCL21, and we observed CCL21^+^; LYVE-1^−^ vessels within BALT that were absent elsewhere in *Ikkα^F/F^* or *Ikkα^Lyve-1^* lungs. It is therefore possible that CCL21^+^; MECA79^−^ HEVs are formed and present in BALT in *Ikkα^Lyve-1^* mice. The MECA79 epitope is created by modifying enzymes that require LTβR signaling for expression, and transcriptomic analysis revealed that HECs in peripheral LNs express low levels of *Lyve-1* (43). Thus, loss of IKKα in LYVE-1^+^ BECs in *Ikkα^Lyve-1^* mice might impact formation of the MECA79 epitope on HEVs in BALT. Alternatively, because BALT forms in the absence of inflammation in *Ikkα^Lyve-1^* mice, the signals required to drive MECA79^+^ HEVs may be absent.

Pioneering studies revealed that iBALT in the lungs of IAV-infected mice contributes to the antiviral immune response (6, 7, 9). Moreover, IAV-driven iBALT develops in mice lacking all secondary lymphoid organs and is sufficient for protective, albeit delayed, immunity in the lung (6, 9). Later studies revealed that preexisting BALT induced in wild-type mice by either neonatal LPS administration or pulmonary instillation of protein nanoparticles provided enhanced protective immunity against IAV and other respiratory viruses (4, 10). Because *Ikkα^Lyve-1^* mice have preexisting nonpathologic BALT without any LNs, we asked if they were similarly protected against IAV infection and found that they had significantly reduced morbidity and mortality compared with *Ikkα^F/F^* littermates. Moreover, *Ikkα^Lyve-1^* mice fully cleared IAV, indicating that the BALT alone is sufficient to mount an antiviral response. Remarkably, viral clearance in *Ikkα^Lyve-1^* mice was no faster than in littermate controls, indicating that the improved morbidity and survival are not due to the existent BALT providing a more rapid or enhanced protective response. We further found that TNF-α, IL-6, and CCL2, which are major drivers of IAV-induced inflammation and immune cell recruitment into the lungs, were unchanged in *Ikkα^Lyve-1^* mice compared with controls, revealing that the initial inflammatory response to the virus was intact. However, a subset of cytokines, most notably IFN-γ, were significantly reduced in *Ikkα^Lyve-1^* mice, leading us to surmise that diminished expression of these and potentially other important immunomodulatory cytokines underlies the improved survival.

CD8 T cells are major effectors of the antiviral immune response and primary producers of IFN-γ following IAV infection (34). CD8 T cells rapidly enter the lungs, where they target IAV-infected cells and play a central role in viral clearance. Consistent with their presence in BALT, the numbers of all T cells were significantly elevated in uninfected *Ikkα^Lyve-1^* mice. However, unlike *Ikkα^F/F^* mice, in which T cells markedly increased following IAV infection, in IAV-infected *Ikkα^Lyve-1^* mice, neither CD4 nor CD8 T cells rose above basal numbers. Most notably, the CD8 population was significantly smaller in IAV-infected *Ikkα^Lyve-1^* lungs than in control mice, indicating an overall deficit in CD8 T cells following infection. These data reveal that T cell migration into IAV-infected lungs is ablated in *Ikkα^Lyve-1^* mice and suggest that fewer numbers of CD8 T cells result in reduced levels of IFN-γ. Moreover, because *Ikkα^Lyve-1^* mice completely clear IAV, the CD8 T cells within the lung before infection appear sufficient to provide full and effective immunity. Reduced T cell entry may reflect the loss of an immune response originating in draining LNs, as suggested for similarly reduced lung T cell numbers in mice lacking all secondary lymphoid organs (6, 9). However, unlike those mice, *Ikkα^Lyve-1^* mice have extensive preexisting BALT; thus, our findings support the concept that this alone provides sufficient anti-IAV immunity. Furthermore, these reduced overall numbers of inflammatory CD8 T cells and decreased IFN-γ levels are likely to contribute to the improved survival of *Ikkα^Lyve-1^* mice.

The precise role of IFN-γ in the immune response to IAV is not fully defined; however, mice lacking IFN-γ or its receptor can efficiently and effectively clear virus (32, 33). Remarkably, similar to our findings with *Ikkα^Lyve-1^* mice, recent work revealed that IFNγ^−/−^ mice exhibit improved morbidity and survival following IAV (PR8) infection (33). Moreover, CD8 T cell–derived IFN-γ was shown to promote monocyte recruitment into IAV-infected lungs driving the disease severity and tissue damage that was reduced in IFNγ^−/−^ mice (33). As expected, IAV infection drove an increase in monocytes in the lungs of *Ikkα^F/F^* and *Ikkα^Lyve-1^* mice from baseline; however, the frequency and number of monocytes was reduced in IAV-infected *Ikkα^Lyve-1^* lung to an extent similar to that previously described in IFNγ^−/−^ mice (33). We found only a marginal reduction in damage in the lungs of IAV-infected *Ikkα^Lyve-1^* mice, and further evaluation of the precise nature, extent, and severity of injury is required. However, together with these prior findings and consistent with reduced CD8 T cell numbers and lower levels of IFN-γ, our experiments suggest that reduced recruitment of monocytes into the lungs of IAV-infected *Ikkα^Lyve-1^* mice supports improved survival.

In conclusion, we have shown that BALT forms spontaneously in *Ikkα^Lyve-1^* mice in the absence of inflammation or tissue damage. Although LYVE-1 is expressed by some BECs and macrophages in the lung (19, 28, 39), our data, together with previous studies of lung lymphatic dysfunction (19), strongly indicate that BALT forms in *Ikkα^Lyve-1^* mice through disruption of the immune homeostatic function of LECs. Importantly we show that preexisting BALT is sufficient to provide virus-clearing immunity to IAV infection and that *Ikkα^Lyve-1^* mice exhibit markedly improved morbidity and mortality. Because the role of lung lymphatics in pulmonary immune homeostasis, respiratory infections, and inflammatory diseases is a rapidly growing area of interest (20), *Ikkα^Lyve-1^* mice are an exciting new model to explore the mechanisms of TLS formation and the immunoregulatory function of lung lymphatics.

## Supplementary Material

Supplemental Material (PDF)
