# Supplementary material for: Loss of Lymphatic IKKα Disrupts Lung Immune Homeostasis, Drives BALT Formation, and Protects against Influenza
Source: Immunohorizons. 2024 Jul 15;8(7):478–91. doi: 10.4049/immunohorizons.2400047 (PMC11294277; doi:10.4049/immunohorizons.2400047)
Supplement: Supplemental Material (PDF) [file IH_2400047_Supplemental_1.pdf]

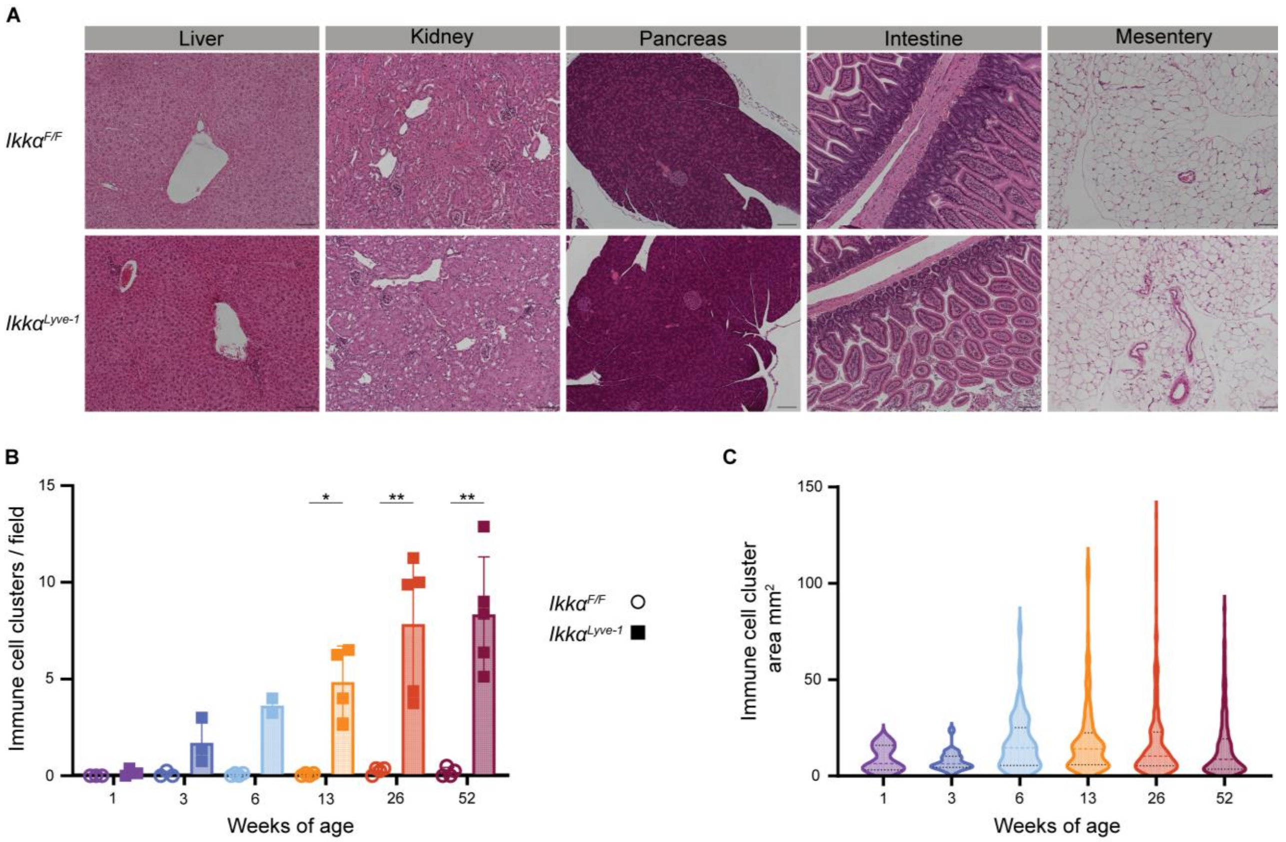

Figure S1. Clusters of immune cells do not appear in other tissues besides the lung in *Ikkα*$^{Lyve-1}$ mice and develop after birth. (**A**) Representative H&E-stained paraffin-embedded tissue sections from *Ikkα*$^{F/F}$ and *Ikkα*$^{Lyve-1}$ mice. Scale bars = 100 μm, 10-weeks of age, n = 5 mice. (**B**) Average number of immune cell clusters within a field of view (5X magnification) of H&E-stained sections of lungs from *Ikkα*$^{F/F}$ and *Ikkα*$^{Lyve-1}$ mice. Statistics, unpaired t test with Welch's correction * $p < 0.05$ ** $p < 0.01$. (**C**) Distribution of size of the immune cell clusters in *Ikkα*$^{Lyve-1}$ mice from (**B**). (**B**-**C**) 1-week of age, n = 3 mice. 3-weeks of age, n = 3 mice. 6-weeks of age, n = 2-3 mice. 13-weeks of age, n = 4 mice. 26-weeks of age, n = 4-5 mice. 52-weeks of age, n = 4-5 mice.

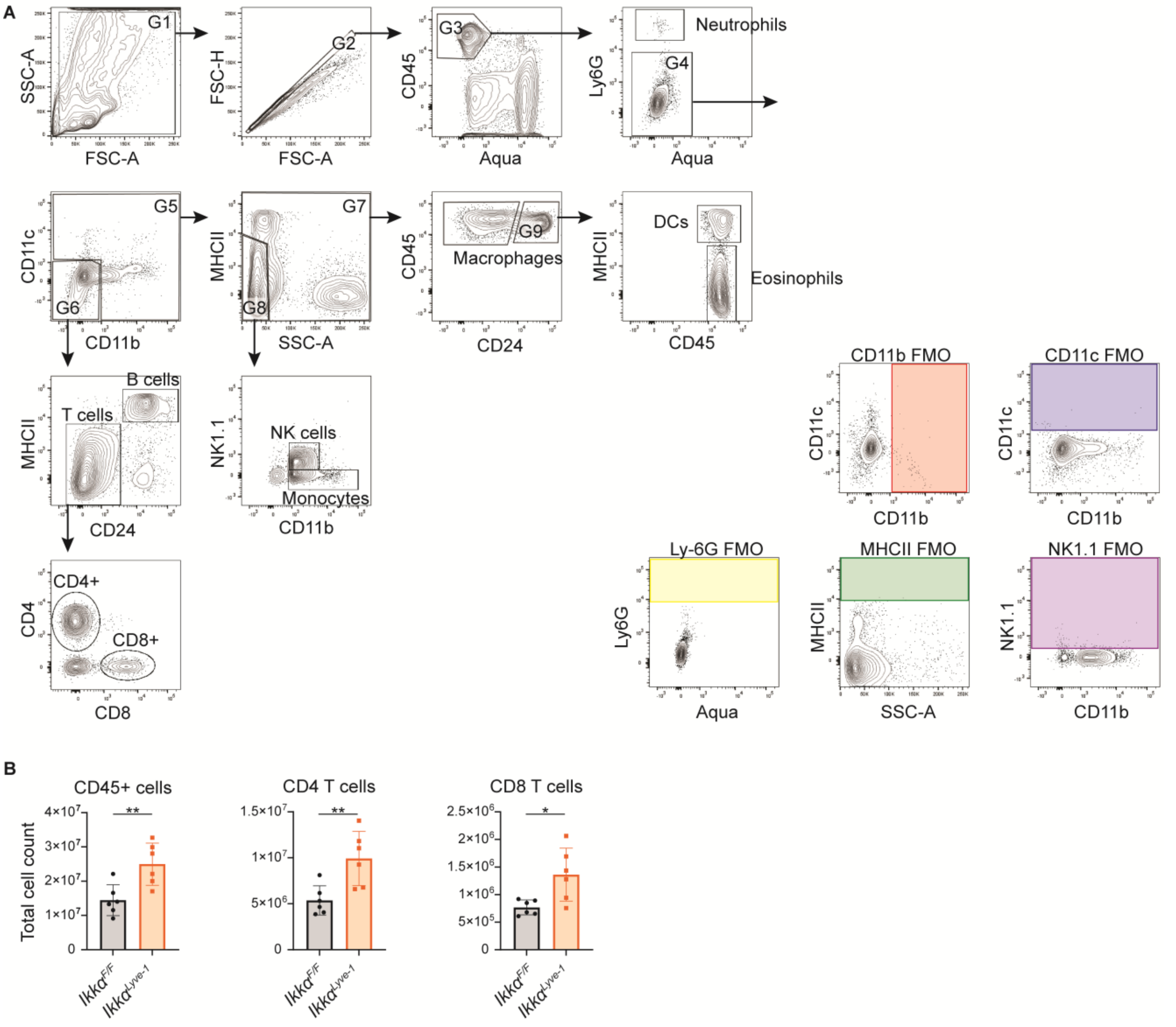

Figure S2. Flow cytometry for immune cell populations in the lung at homeostasis. (**A**) Flow cytometry gating strategy for immune cells at homeostasis. (**B**) Flow cytometry results for CD45+ cells, CD4 T cells, and CD8 T cells. (**A**-**B**) Cells were stained from perfused, homogenized lungs of *Ikkα*$^{F/F}$ and *Ikkα*$^{Lyve\text{-}1}$ mice. 10-weeks of age, $n = 6$. Statistics, unpaired t test * $p < 0.05$ ** $p < 0.01$.

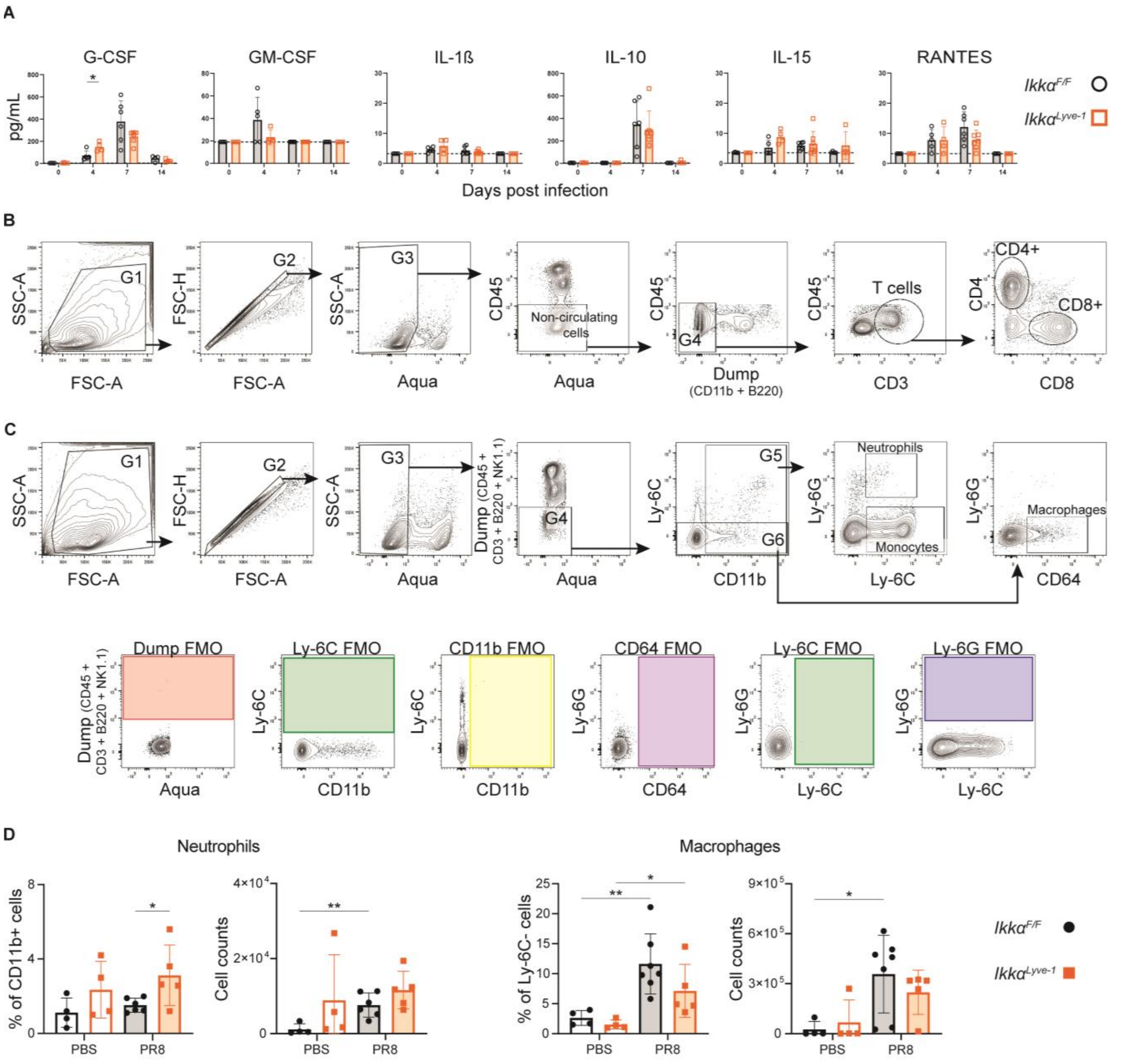

Figure S3. Cytokines and flow cytometry of immune cells from influenza infected lungs. (**A**) Level of cytokines and chemokines in BALF throughout influenza infection. n = 4-7 mice. Statistics, unpaired t test. Data represents the mean ± SD. (**B**) Flow cytometry gating strategy for T cells. (**C**) Flow cytometry gating strategy for monocytes, neutrophils, and macrophages. (**B**-**C**) Cells were stained from homogenized lungs on day 7 post influenza infection. Mice were injected with anti-CD45 Ab to label circulating cells prior to euthanasia. (**D**) Flow cytometry results for neutrophils and macrophages on Day 7 following PBS or influenza PR8 treatment. n = 4-7 mice. Outliers were tested for using the ROUT method with a Q = 1%. Statistics, unpaired t test * p < 0.05 ** p < 0.01.

Table SI: *p* values from comparing *Ikkα*$^{F/F}$ and *Ikkα*$^{Lyve-1}$ analyte levels in BALF[a].

Analyte	Day post infection			
	0	4	7	14
IL-2	ND	ND	ND	ND
IL-4	ND	ND	ND	ND
IL-7	ND	ND	ND	ND
IL-12 (p40)	ND	ND	ND	ND
Il-12 (p70)	ND	ND	ND	ND
IL-13	ND	ND	ND	ND
IL-17	ND	ND	ND	ND
IL-19	ND	ND	ND	ND
MIP-2	ND	ND	ND	ND
GM-CSF	ND	0.1929	ND	ND
IL-1β	ND	0.2536	0.2757	ND
IL-10	0.6395	0.2924	0.6922	0.3559
IL-15	0.3559	0.0629	0.7033	0.3567
RANTES	ND	0.9933	0.0904	ND
TNFα	ND	0.6034	0.0807	0.5832
IL-6	0.3559	0.9244	0.9113	0.2507
CCL2	ND	0.2298	0.0817	0.1425
CXCL1	0.2333	0.3344	0.3675	0.1111
CXCL10	0.1879	0.5437	0.0847	0.2219
G-CSF	0.4491	0.0311	0.1001	0.2460
IL-1α	0.9466	0.0273	0.0737	ND
IL-5	ND	0.2574	0.0050	0.9449
CCL3 (MIP-1α)	ND	0.0354	0.0013	ND
CCL4 (MIP-1β)	ND	0.4416	0.0177	0.2336
IFNγ	ND	0.1923	0.0435	ND

[a] This table shows the *p* values from comparing the level of analytes in BALF between *Ikkα*$^{F/F}$ and *Ikkα*$^{Lyve-1}$ mice at time points throughout IAV infection. Analytes that were not detected in BALF for either *Ikkα*$^{F/F}$ and *Ikkα*$^{Lyve-1}$ mice are indicated by "ND." Comparisons were made using a 2-tailed unpaired *t* test.
